# Impact of artisanal refining activities on bacterial diversity in a Niger Delta fallow land

**DOI:** 10.1038/s41598-024-53147-4

**Published:** 2024-02-16

**Authors:** Emmanuel Oliver Fenibo, Rosina Nkuna, Tonderayi Matambo

**Affiliations:** 1 World Bank Africa Centre of Excellence for Oilfield Chemical Research, University of Port Harcourt, Choba, Rivers State Nigeria; 2 Department of Biotechnology, Faculty of Applied and Computer Sciences, Vaal University of Technology, Vanderbijlpark 1900, Gauteng, South Africa; 3 Centre for Competence in Environmental Biotechnology, College of Animal and Environmental Science, University of South Africa, Florida Science Campus, Roodepoort, South Africa

**Keywords:** Biochemistry, Biotechnology, Computational biology and bioinformatics, Ecology, Genetics, Microbiology, Molecular biology, Ecology, Environmental sciences

## Abstract

Hydrocarbon pollution is a major ecological problem facing oil-producing countries, especially in the Niger Delta region of Nigeria. In this study, a site that had been previously polluted by artisanal refining activity was investigated using 16S rRNA Illumina high-throughput sequencing technology and bioinformatics tools. These were used to investigate the bacterial diversity in soil with varying degrees of contamination, determined with a gas chromatography-flame ionization detector (GC-FID). Soil samples were collected from a heavily polluted (HP), mildly polluted (MP), and unpolluted (control sample, CS) portion of the study site. DNA was extracted using the Zymo Research (ZR) Fungi/Bacteria DNA MiniPrep kit, followed by PCR amplification and agarose gel electrophoresis. The microbiome was characterized based on the V3 and V4 hypervariable regions of the 16S rRNA gene. QIIME (Quantitative Insights Into Microbial Ecology) 2 software was used to analyse the sequence data. The final data set covered 20,640 demultiplexed high-quality reads and a total of 160 filtered bacterial OTUs. Proteobacteria dominated samples HP and CS, while Actinobacteria dominated sample MP. *Denitratisoma*, *Pseudorhodoplanes*, and *Spirilospora* were the leading genera in samples HP, CS, and MP respectively. Diversity analysis indicated that CS [with 25.98 ppm of total petroleum hydrocarbon (TPH)] is more diverse than HP (with 490,630 ppm of TPH) and MP (with 5398 ppm of TPH). A functional prediction study revealed that six functional modules dominated the dataset, with metabolism covering up to 70%, and 11 metabolic pathways. This study demonstrates that a higher hydrocarbon concentration in soil adversely impacts microbial diversity, creating a narrow bacterial diversity dominated by hydrocarbon-degrading species, in addition to the obvious land and ecosystem degradation caused by artisanal refining activities. Overall, the artisanal refining business is significantly driving ecosystem services losses in the Niger Delta, which calls for urgent intervention, with focus on bioremediation.

## Introduction

The Niger Delta, located in Nigeria, is a well-researched area in Africa, renowned for its potential for oil exploration and its diverse ecosystem. It spans 70,000 km^2^, making it the largest wetland in Africa and the third-largest in the world^1^. Furthermore, it holds the most substantial oil reserves (36.2 billion barrels) and gas reserves (185 billion cubic feet) in Africa^2^. The history of commercial oil and gas exploration in the Niger Delta goes back to 1958, which has led to an estimated 3.1 billion barrels of crude oil being discharged, affecting over 5000 sites from 1967 to 2014. This has resulted in the region being identified as one of the most polluted areas globally^3–5^. Studies have shown that oil pollution in the region is attributed to inadequate servicing and maintenance facilities, routine oil operations, sabotage, accidental/equipment failures, deliberate release of oil wastes into the environment, and currently, artisanal oil refining activities^6–8^. Petroleum pollution releases various types of hydrocarbons (saturates, aromatics, resins, and asphaltenes) into the environment, ultimately impacting the terrestrial ecosystem. This impact arises from their ability to disrupt the healthy ecological balance^9^, terminating sensitive ecological receptor^10^, contaminate groundwater, pose health challenges^10^, as well as reduce, microbial composition^11,12^. The shift in microbial structure favours sentinel microorganisms that metabolize those complex hydrocarbons as sources of carbon and energy for growth and other physiological processes^13,14^.

Petroleum hydrocarbon-degrading bacteria are abundant in contaminated habitats^15,16^. Autochthonous bacteria with this specialty are ubiquitous in the terrestrial ecosystem and are key to the natural attenuation and detoxification of such impacted lands^17^. Shen et al.^18^ evaluated the bacterial diversity of polluted soil by means of metagenomics and confirmed the dominance of Actinobacteria. A diversity study conducted by Obieze et al.^19^ demonstrated that populations of hydrocarbon-utilizing bacteria were highest when the concentration of pollutants were high. Chikere et al.^20^ used 16S rRNA technology to show that the shift of bacterial genera moved from a mixed group to Gram-negative bacteria with Betaproteobacteria dominance.

Bacterial diversity studies on terrestrial ecosystem impacted by artisanal crude oil refining are an emerging area in the Niger Delta. This necessitates MiSeq-driven metagenomics, which is a reliable Next-Generation Sequencing (NGS) technology for evaluating bacterial compositional structure, diversity, and function. In this study, we investigated bacterial diversity insights in polluted soil using MiSeq sequencing technology to understand how varying hydrocarbon concentrations affect the distribution of bacteria.

## Results

### Physicochemical analysis

The analysis of soil texture indicated that gravel constituted 5.2%, sand comprised 93.2%, clay constituted 4.4%, and silt accounted for 2.4%. Various chemical parameters, encompassing pH, electrical conductivity, total nitrogen, and total organic carbon, were evaluated for both the contaminated soil and control soil samples, with the results summarized in Table 1. The pH values for the control, mildly polluted, and heavily polluted soils were recorded as 6.50, 6.30, and 7.10, respectively. The electrical conductivity (EC) values for the control, mildly polluted, and heavily polluted soils were 30 µS/cm, 40 µS/cm, and 120 µS/cm, respectively. Similarly, the total nitrogen values were 2.67 mg/kg, 2.02 mg/kg, and 2.00 mg/kg for the control, mildly polluted, and heavily polluted soils, respectively. Additionally, the total organic carbon was 1.25%, 5.25%, and 19.61% for the control, mildly polluted, and heavily polluted soils, respectively.

**Table 1:** Physicochemical characteristics of the control and polluted soil sample.

Physicochemical characteristics	Heavily polluted soil (HP)	Control soil (CS)	Mildly polluted soil (MP)
Soil type	Loamy sandy	Loamy sandy	Loamy sandy
pH	6.50	6.30	7.10
Electrical conductivity (µS/cm)	30	40	120
Total nitrogen (mg/kg)	2.67	2.00	2.02
Total organic carbon (%)	1.25	5.25	19.61
TPH (ppm)	490,630	25.98	5398

### TPH analysis

The values of the TPH for the heavily polluted (HP), control sample (CS) and mildly polluted (MP) soil sample are 490,630, 25.98 ppm, and 5398 ppm respectively.

### Bacterial diversities from unpolluted, mildly polluted and highly polluted soils

**Figure 1:**
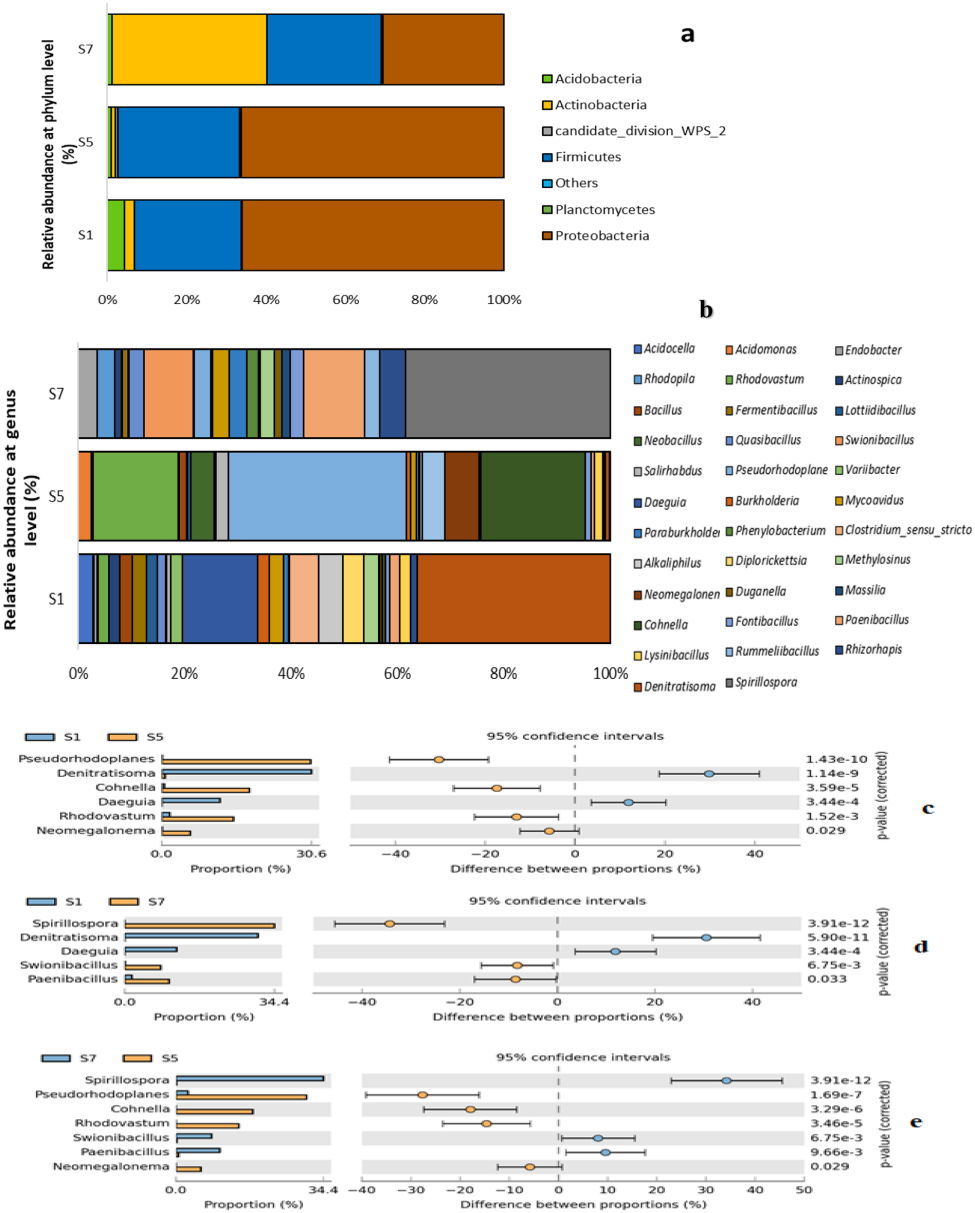
Relative abundance of bacteria. Relative abundance at phylum (**a**) and genus level (**b**). An extended error bar plot was used to compare the top 10 genera between two samples with p < 0.05 as shown: S1 and S5 (**c**); S1 and S7 (**d**) and S7 and S5 (**e**). *S1* Heavily polluted (HP), *S5* Control sample (CS), *S7* Mildly polluted (MP).

The final dataset from the three reference samples—heavily polluted (HP), unpolluted (control sample; CS), and mildly polluted (MP)—comprised 20,640 demultiplexed high-quality reads, with an average of 6880 reads per sample. The sequences were clustered into 256 bacterial OTUs, which were further filtered to a minimum count of 4 and 20% prevalence per sample, resulting in a total of 160 retained bacterial OTUs. The dominant bacterial phyla in the heavily polluted soil (HP) samples were Proteobacteria (66%), Firmicutes (27%), Acidobacteria (4%), and Actinobacteria at less than 3%. In the unpolluted soil (CS) sample, the dominant phyla were Proteobacteria (68%), Firmicutes (30%), and Actinobacteria at less than 2%. In the mildly polluted soil (MP), the dominant phyla were Actinobacteria (39%), Proteobacteria (31%), Firmicutes (29%), Acidobacteria (0.67%), and Planctomycetes (0.33%). Figure 1 summarises the bacterial phylum distribution in the three reference points. Dominant genera in the HP sample are *Denitratisoma*, *Clostridium*, *Alkaliphilus*, *Diplorickettsia*, *Methylosinus* and *Bacillus* (in descending order: ranging from 30.56% to 1.99%)*.* The following genera (*Pseudorhodoplanes*, *Cohnella*, *Rhodovastum*, *Neobacillus*, *Neomegalonema*, *Acidomonas*, *Neobacillus*, *Salirhabdus*) are duplicated in HP and CS samples, however, at relatively low abundance (< 2.0%) in HP but was dominant genera in CS. These genera are not observed in MP, except for *Pseudorhodoplanes* (2.7%). The dominant bacterial genera in MP include *Spirilospora* (34%), *Paenibacillus*, *Swionibacillus*, *Rhizorhapis*, *Endobacter*, *Paraburkholderia*, *Rhodopila*, *Mycoavidus*, *Rummeliibacillus*, *Quasibacillus*, and *Phenylobacterium*. Genera such as *Denitratisoma*, *Alkaliphilus*, *Clostridium*, and *Bacillus* were observed in CS but not in MP samples, while *Methylosinus* was observed in MP and not in CS. Figure 1a shows the dominant phyla diversity across the three samples, while Fig. 1b displays the relative abundance of the ten most dominant genera. Figures 1c–e depict that the bacterial abundance in CS is significantly different from both HP and MP (at *p* = 0.05). Similarly, as displayed in Table 2, CS is higher in richness and diversity as compared to the HP and MP samples.

**Table 2:** Statistical estimate of bacterial abundance and diversity index in the TPH-gradient soil samples.

Sample	TPH concentration	Sequence	OTU number	Observed OTU	Shannon Index
HP	490,630 ppm	74,048	399	10	1.1206
CS	25.98 ppm	53,118	1314	11	1.3363
MP	5398 ppm	50,912	644	9	1.1673

### Inferred bacterial function by PICRUSt2

**Figure 2:**
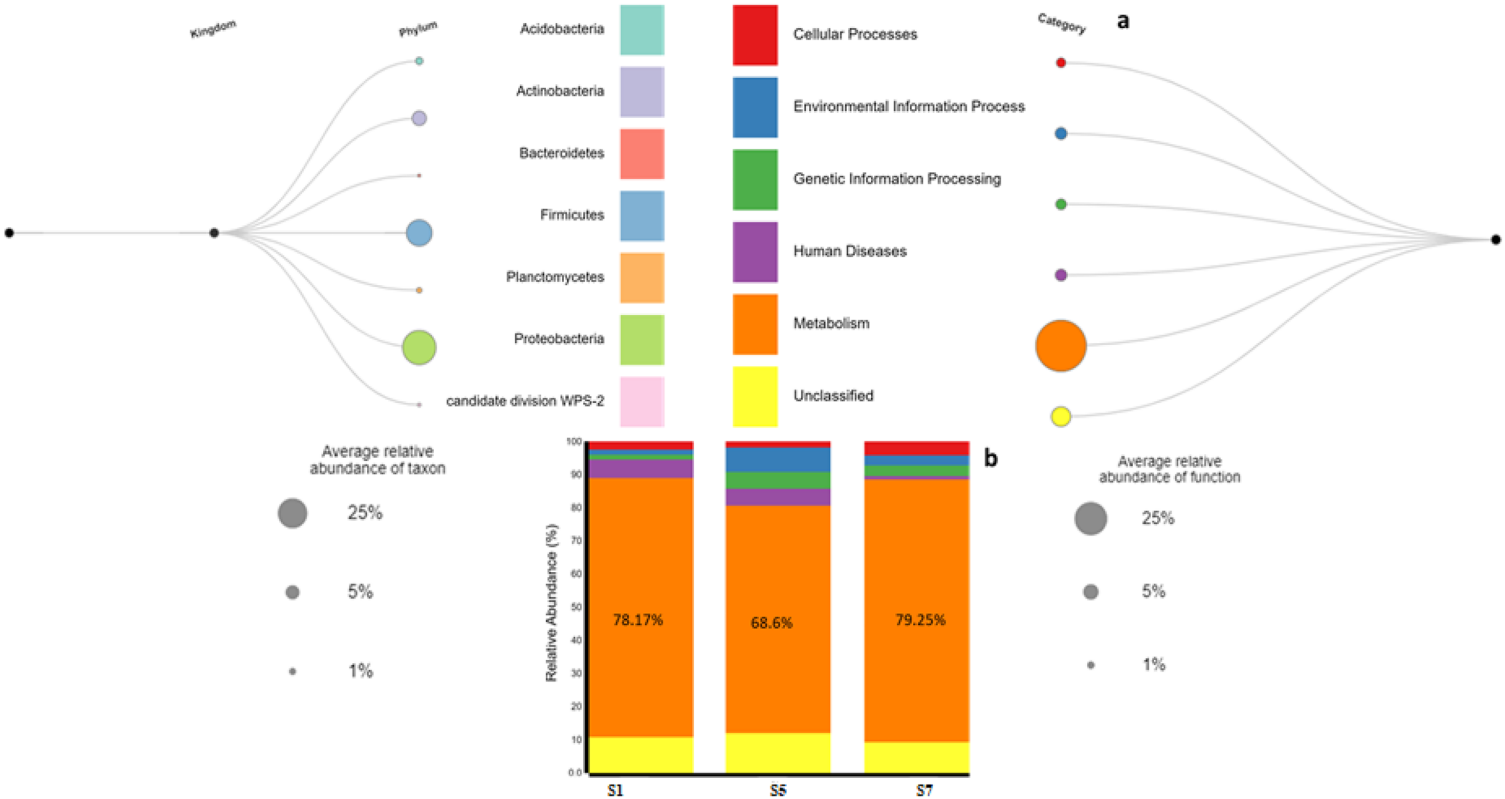
The link between taxonomy and predicted function. (**a**) Shows the abundance of predicted function and phylum. (**b**) Shows the relative abundance of each category of the predicted function. BURRITO, a visualization tool for exploratory data analysis of metagenomic data was used to visualize taxonomy linked to predicted function. *S1* Heavily polluted (HP), *S5* Control sample (CS), *S7* Mildly polluted (MP).

**Figure 3:**
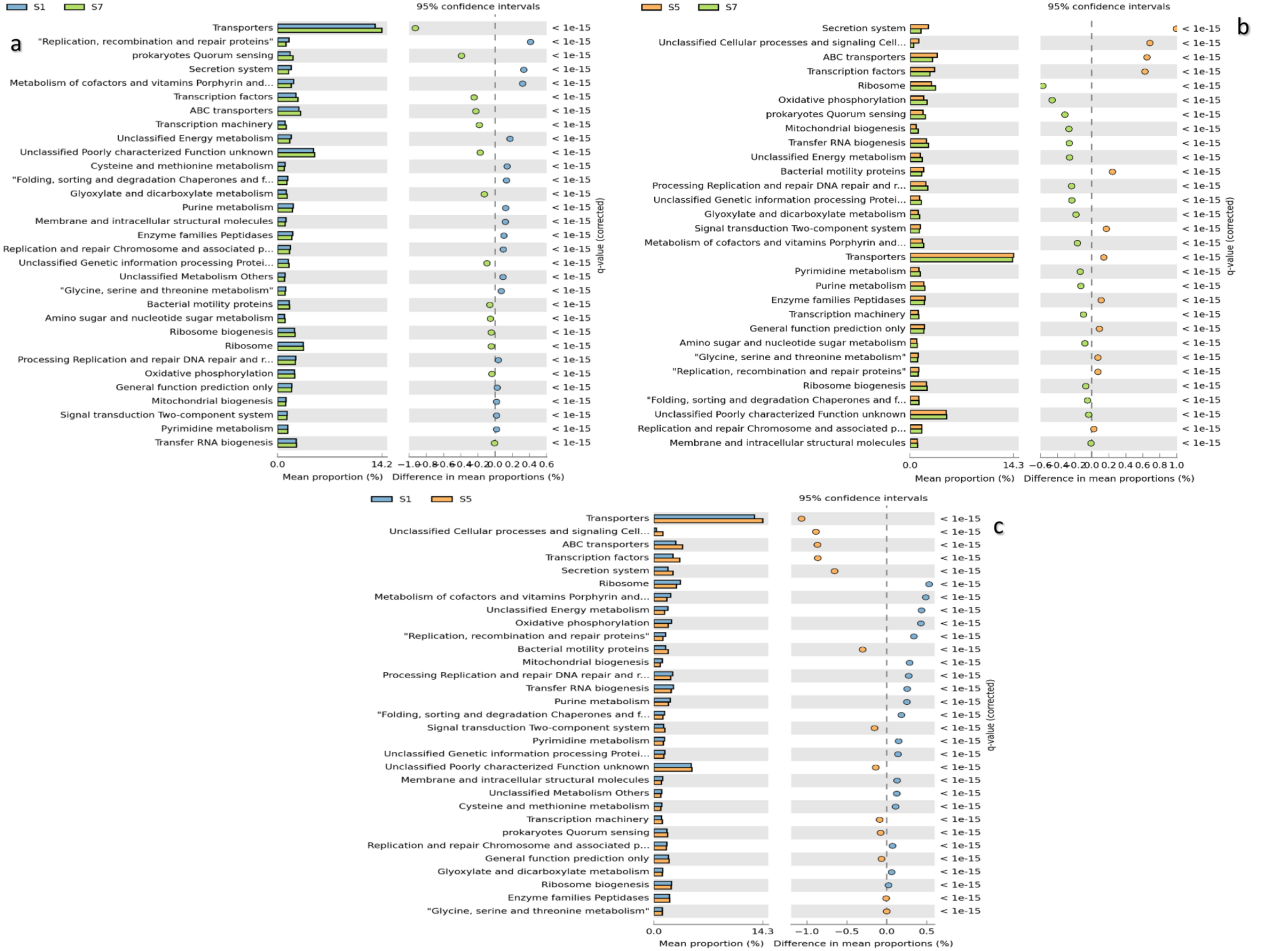
An overall comparison of KEGG metabolic sub-pathway of the category metabolism for top three phyla (Proteobacteria, Firmicutes and Actinobacteria) as inferred by PICRUSt2. (**a**–**c**) depict significant (q-value > 0.05) features between two groups, calculated using White’s non-parametric test with Benjamini–Hochberg FDR (false discovery rate) in STAMP. *S1* Heavily polluted (HP), *S5* Control sample (CS), *S7* Mildly polluted (MP).

A total of 99 KOs (KEGG Orthologues) were predicted, and the superpathway was used for the plot. The predicted KEGG (Kyoto Encyclopedia of Genes and Genomes) orthologues, collapsed into MetaCyc meta-pathways, show abundance values for each sample using the PICRUSt methodology (Fig. 2). Six functional modules, comprising cellular processes (3%), environmental information processing (7%), genetic information processing (5%), human diseases (5%), and metabolism (70%), make up almost 90% of the complete dataset in the samples. The higher percentage of metabolism is registered in the order of MP, HP and CS respectively, indicating that hydrocarbon serves as a source of carbon or energy or both. Differentially abundant pathways (at p ≤ 0.05) showed 11 pathways (Fig. 3), with oxidative phosphorylation as the most dominant biomarker pathway. Heavily polluted (HP) sample is more pronounced with disease function, with a notable genus such as *Diplorickettsia*, an agent of tick-borne infection. CS leads in genetic information processing, while MP leads in cellular processes.

## Discussion

Total petroleum hydrocarbons (TPH) consist of various fractions of petroleum, including polyaromatic hydrocarbons (PAHs). TPH in soil is primarily derived from anthropogenic sources, but some fractions of PAHs are contributed by living organisms such as plants and microorganisms^21^. The major contribution of TPH in the study site was artisanal activities, which include spills, explosions, combustion, deposition, and the burial of the heavy fraction regarded as waste. Intrinsic factors such as soil texture, temperature, biosynthesis, topography, and erosion contribute to the overall concentration of soil TPH. The last two factors play an influential role in the unequal distribution of TPH in the soil. The concentration of the control sample (25.98 ppm) is well below the 100 ppm threshold required as a clean-up standard^22^. The mildly polluted soil (with value of 5398 ppm) and heavily polluted soil (with a value of 490,360 ppm) is quite significant and is comparable to that TPH concentration obtained by Martinez et al.^23^. The mildly polluted soil TPH concentration exceeds the Nigerian regulatory standard, 5000 ppm^24^. The artisanal refining operations in the Niger Delta have a profound impact on the environment as a whole. The pollution that originates from artisanal refining sites is characterized by extremely poor air quality^25^, contamination of surface soil and groundwater^26^, loss of vegetation and mangroves^27^ and heavy pollution of the marine ecosystem^28^. The air, consistently laden with hydrocarbons, has the potential to cause and exacerbate respiratory diseases^29^ such as reduced lung function, bronchitis, asthma, lung cancer, and chronic obstructive pulmonary disease^30–32^. The contamination of surface soil from artisanal refineries leads to a significant loss of vegetation cover and arable land, as well as a drastic alteration of microbial diversity. Contaminated groundwater poses a risk to both animals and humans, serving as a source of unsafe and toxic water. The disposal of the heavy-end fraction of hydrocarbons and other affiliated wastes (as is usually the practice) in water bodies remains a crude method of waste management. Consequently, the marine ecosystem becomes heavily polluted, resulting in a severe loss of mangrove habitats^33,34^. Seafood from the marine ecosystem is affected, leading to bioaccumulation and biomagnification^35,36^, which ultimately results in cancer and other malignant growths in humans. Overall, artisanal refining activities lead to health challenges, decreased agricultural productivity, reduction in the means of livelihood in local communities, and significant losses in biodiversity and ecosystem services, implicating microorganisms.

Microorganisms react to various situations of hydrocarbon pollution in different ways: they either develop resistance or utilize hydrocarbons as a source of carbon and energy. Those that are unable to adapt to the stress of hydrocarbon pollution are eliminated^12^. In all the samples analysed, there were changes in the structure of biodiversity compared to the unpolluted control. The dynamics of the phylum shift, reflecting the dominance of Proteobacteria in the heavily polluted (HP) and control sample (CS) soil samples and not in the mildly polluted (MP) is similar to those observed in the study conducted by Kim et al.^37^. The Firmicutes did not respond significantly to each of the samples as did Proteobacteria, Actinobacteria, and Acidobacteria. The dominance of Proteobacteria in both heavily polluted and unpolluted soils accounts for the relevant roles they play in the biogeochemical cycling of carbon, sulphur, and nitrogen^38^, as well as plant fitness and growth promotion^39^. The shift noticed in the mildly polluted sample favoured the dominance of Actinobacteria. Genera in this group thrive in hydrocarbon-polluted environment, highlighting their physiological and genomic adaptations to challenging conditions^40^. These bacteria play a crucial role in diverse ecological processes, such as the biodegradation of complex molecules^41^, involvement in biogeochemical cycles^42^, deterioration of artefacts, and participation in biological weathering^43^. Actinobacteria demonstrate proficiency in these functions owing to their exceptional capabilities in DNA repair and protection, protein synthesis, biofilm formation, as well as the synthesis of biosurfactants, secondary metabolites, and essential enzymes^44–47^. Notably, certain genera such as *Geodermatophilus*, *Modestobacter*, and *Kocuria*, within the Actinobacteria phylum, exhibit resistance to desiccation, heavy metal toxicity, and ionization, as reported by Sayed et al.^48^, Shivlata and Satyanarayana^49^ and Guesmi et al.^50^ Consequently, Actinobacteria represent a valuable resource for the bioremediation of highly contaminated environments. The trend shows that Acidobacteria diversities decreased significantly in the mildly polluted soil. This study demonstrates that hydrocarbon contamination gradients influence Proteobacteria, Acidobacteria and Actinobacteria as Abena et al.^51^ showed.

The prevalent genera identified in the heavily polluted (HP) sample include *Clostridium* (Firmicutes), *Methylosinus* (Alphaproteobacterial methanotroph), and *Bacillus* (Firmicutes) among other genera including *Denitratisoma* (Betaproteobacteria) and *Daegula* (Alphaproteobacteria). The last two genera are the two most dominant in the HP sample. *Clostridium* species have the requisite enzyme system^52^, genetic repertoire^9,53^ and cell surface properties^54^, to access and degrade hydrocarbon (especially halogenated species) under anaerobic condition. *Methylosinus* is a methanotrophic bacteria that has proven proficiency in degrading methane and chlorinated hydrocarbon^55,56^ in consortium with other hydrocarbon-degrading bacteria and communities^57^. *Bacillus* spp. is a widely distributed prolific bacteria known for their ability to metabolise hydrocarbon taking advantage of their ability to form biofilm^58^, genomic capacity^59^, biosurfactant production^60^, metabolic diversity^61^, and favourable redox potentials^62^. Some species are hydrocarbonoclastic^63^, thus effecting an increased population density as they utilise hydrocarbon as source of energy and carbon. While *Denitratisoma* and *Daegula* exhibit the highest population density in the heavily polluted soil, there is limited information available about them. However, it is established that *Denitratisoma* functions as aerobic denitrifiers^64^ and plays a crucial role in rhizoremediation^65^. Their reduced presence in the mildly and unpolluted polluted soil suggest that they may have affinity for highly polluted environment. Genera like *Neomegalonema*, *Neobacillus*, *Acidomonas*, *Pseudorhodoplanes*, *Cohnella*, *Rhodovastum*, and *Salirhabdus*, each comprising less than 2% in the highly polluted sample, exhibited high abundance in the unpolluted sample, indicating their sensitivity to hydrocarbon pollution. However, these genera did not show in the mildly polluted soil, except *Pseudorhodoplanes.* This suggests that *Pseudorhodoplanes* is an excellent hydrocarbon-degrading bacteria or an emerging hydrocarbonoclastic bacteria. This bacteria genus has been implicated in hydrocarbon degradation^12^. Tirandaz et al.^66^ illustrated that *P*. *sinuspersici* exhibits optimal activity at a temperature of 30 °C and a pH of 7. However, it demonstrates tolerance within a pH range of 5.5 to 8 and a temperature range of 15 to 35 °C. It is interesting to note that the study site reflects these optimal parameters, including suitable soil (sandy loamy) type. Other genera that appeared prominent in the unpolluted control sample that has been reported to affiliate with hydrocarbon polluted soil are *Cohnella*^67^, *Rhodovastum*^68^ and *Salirhabdus*^69^. Genera that shifted from being rare in the unpolluted sample to becoming more prominent in the polluted samples can be considered as emerging hydrocarbon-degrading bacteria (or tolerant taxa), and they include *Spirilospora*, *Swionibacillus*, and *Paenibacillus*.

The catalogue of hydrocarbon-degrading bacteria is abundant with genera like *Paenibacillus*, *Paraburkholderia*, *Methylosinus*, and *Phenylobacterium*. In the mildly polluted soil examined in this study, these four genera are present, along with the identification of less common bacterial genera, including *Spirilospora*, which appears to be the most prevalent bacterial genus. *Paenibacillus* spp. (also abundant in the heavily polluted sample) has been shown to degrade hydrocarbon in consortium with *Gordonia*, *Cupriavidus* spp^70^., is associated with rhizoremediation, produce biosurfactants in contaminated soil, harbour hydrocarbon-degrading *pahE* genes and other requisite genes^71^. These key biomarkers and other biological factors have positioned *Paenibacillus* spp. to degrade PAH^72^ and transform heavy crude to light oil^73^. Kanwal et al.^74^ had indicated that sporogeneisis permits *Paenibacillus*, *Bacillus* and other related bacteria to survive inhospitable environments. Some species of *Paenibacillus* has been described as hydrocarbonoclastic^75^ and at the same time diazotrophic^76^ in the total environment, highlighting their dual relevance in bioremediation and plant growth promotion. Diazotrophic *Paenibacillus polymyxa* has been implicated in hydrocarbon degradation^77^ specifically polyaromatic hydrocarbon^78^ and biodegradation of mixed pesticides^79^. However, there are more diazostrophic *Paenibacillus* spp. than hydrocarbon-degrading *diazotrophic Paenibacillus* spp. confirmed by publicly available literature^80–82^. From the key hydrocarbon-degrading bacteria examined, it is worthy of note that hydrocarbon degradation is linked to biological factors such as adaptation, metabolic competence, genetics, enzyme system, biomass, biosurfactant production, cell surface property, microbial interaction, biofilm formation and cell’s redox potential. Activation of a considerable number of these attributes has defined *Paraburkholderia*, *Methylosinus*, and *Phenylobacterium* as hydrocarbon-degrading bacteria and possibly *Spirilospora* and *Pseudorhodoplanes*, noted as emerging hydrocarbon-degrading bacteria^83–85^. *Paraburkholderia aromaticivorans* BN5 has been reported to degrade aliphatic hydrocarbons, naphthalene and BTEX^83^, while *Methylosinus* spp. is an obligate methane metabolizer^86^ apart from degrading hydrocarbon through cometabolic pathway^57^. In addition, *Methylosinus* has species that are diazotrophic^87^, and heavy metal detoxifiers. For its part *Phenylobacterium* spp. has been reported in PAH degradation^88^. Signature sequences in the mildly polluted soil is an indication of hydrocarbon metabolism, some of which may represent emerging hydrocarbon-degraders while some may be affiliated with diazotrophism. The diversity of these signature sequences is critical to the understanding of their structure in relation to the heavily polluted (HP) and control (CS) soil samples.

The OTU’s diversity index shows that CS is higher in richness (by observed OTUs), while the Shannon diversity index shows that the CS sample is higher in diversity. The higher diversity index in CS reflects the unpolluted nature of the control sample and the toxicity effect of obvious pollution in HP and MP samples. The consequence of hydrocarbon impact include the shift of broad microbial diversity characterised with broad ecological functions to a narrow microbial diversity with prominent hydrocarbon-degrading and hydrocarbon-tolerant phylotypes. Examination of the mildly and heavily polluted samples reflect a few number of notable and emerging hydrocarbon-degrading bacteria including *Paenibacillus*, *Paraburkholderia*, *Methylosinus Phenylobacterium*, *Bacillus*, *Burkholderia*, *Alkaliphilus*, *Pseudorhodoplanes* and others. The reason behind this “shift to the left’ phenomenon is the adaptation of keystone species to survive in a stressed ecosystem either as resistant or utilizers of hydrocarbon as source of carbon and energy. Bacteria that can tolerate and degrade moderate hydrocarbons, in their mixed form, can initiate biodegradation process, as much as those that can tolerate and benefit from the process’ metabolites^89^. This phenomenon becomes prominent in ecosystem with long-term history of pollution^90^. Results from most studies in pollution ecology and bioremediation align with the concept of broad-to-narrow concept, confirmed in this study. The trend observed in this study counters that of Benedek et al.^91^ which observed positive correlation between TPH (147,000 ppm) and diversity. The reasons, according to Benedek et al.^91^, for this negative results are long-term exposure to hydrocarbon, significant rise of a particular hydrocarbon-degrading bacterial genus, need for alternative carbon source and lack of humus. Recent studies that support positive correlation between TPH and microbial diversity in the Niger Delta are Iturbe-Espinoza et al.^90^, Edet and Antai^92^ and in other regional settings are Lee et al.^93^, Mukherjee et al.^94^ and Yerulker et al.^95^. These two contradicting research outcome suggests that besides, ecotoxicity effects, other factors play influential roles in diversity profiling^96^. These factors may include soil chemistry, soil’s trophic status, and genera composition in higher taxa. These factors condition a non-uniform trend and responses of microbial taxa to contamination in soil.

In pollution-affiliated microbial diversity dynamics, dominance of hydrocarbon-degrading bacteria is a common phenomenon, which is underscored by biological functions such as cellular processes, genetic information and degradation. Hydrocarbons are utilised as an energy source through oxidative phosphorylation, in the inner section of bacteria cytoplasm with the release of ATP for cellular processes: growth, replication, quorum sensing, chemotaxis, and catabolism. The latter is achieved through the use of key signatory enzymes. Though an enzyme profile was not conducted in this study, Obieze et al.^19^ reported hydrocarbon-degrading enzymes (through functional prediction) in the same study site, which include (3S,4R)-3,4-dichloroxycyclohexa-1,5-diene-1,4-dicarboxylate dehydrogenase, 2,4-diclorophenol-6-monooxygenase, 3-carboxyethylcatechol-2,3-dioxygenase. Bidja-Abena et al.^51^ identified a few functional enzymes (in diesel-polluted soil) specific for xenobiotic metabolisms, chlorobenzene degradation, and polyaromatic hydrocarbon. Protein export and gene repair (indices of genetic information processing) are protective against hydrocarbon toxicity to cells^97,98^. Another KEGG functional profile that is connected to metabolism is environmental information processing such as the ABC transporters for mineral/organic ions, amino acids, and lipid transportation^38^. Protein transportation is necessary for uptake of hydrocarbons by bacteria for degradation and metabolism. These functional module is dominated by metabolism with 11 pathways, which support active engagement of hydrocarbon degradation in this study.

## Concluding remarks

This work was undertaken to understand the impact of artisanal refining activities on soil bacterial diversity through metagenomics. Consequently, Ngia Ama was chosen because of its hydrocarbon pollution history of more than six years. Composite samples of heavily (HP) polluted, mildly (MP) polluted and unpolluted (CS) soil were used for the analysis and the results showed a broad-to-narrow bacterial diversity with known and emerging hydrocarbon-degrading bacteria found abundantly in polluted samples. This paradigm shifts in bacteria diversity points to distortion of ecological service at the detriment of the total environmental and its receptors in addition to the vicious impact of artisanal refining activities on vegetation and marine ecosystem in the Niger Delta. However, the study area features as a hub of activated soil critical for ex situ bioremediation programme.

## Methodology

### Site description

The study was carried out on contaminated fallow land at Ngia Ama (4°47ʹ42ʺ N, 6°51ʹ45ʺE), a community in Tombia Kingdom where illegal refining activities had occurred for over six years, with respect to the sampling year, 2018. The study site is enveloped by mangroves and creeks, featuring moderate lowlands and an average temperature of 25–37 °C.

### Soil sample collection

The heavily (HP) and moderately (MP) polluted portions (7.5 ft away from each other) of the study site were spotted and soil sampling was carried out using a soil auger. Three sub-samples were collected at each point from a depth of ≤ 30 cm and mixed thoroughly to create a composite sample. A similar approach was used to collect a control sample (CS, non-polluted), 23 ft away from the polluted field. The samples were aseptically transferred into sterile plastic containers and preserved at 4 °C and − 20 °C for downstream analysis.

### Soil’s physicochemical analysis

The soil samples collected were initially dried at 25 °C, ground, and then sieved through a 2 mm mesh before analysis, following the method outlined by Durak et al.^99^. The Bouyoucos Hydrometer method was employed for soil texture analysis, following the protocol adopted by Babalola et al.^100^, to determine the content of sand, silt, and clay. In summary, a beaker was filled with 50 g of pre-treated soil and 125 ml of sodium hexametaphosphate (40 g/L) was added. The mixture was stirred until the soil was fully saturated and then left to rest for ten minutes. The resulting soil slurry was moved to a mixer, and distilled water was added until the mixing cup was half full. The solution was then mixed for two minutes. Subsequently, the soil slurry was quickly transferred to an unoccupied sedimentation cylinder, and distilled water was added up to the reference mark. The cylinder was flipped upside down and back 30 times. After placing the cylinder down, the time was noted. The stopper was taken off the cylinder, and a hydrometer was gently inserted. The initial hydrometer reading was taken immediately, followed by a second reading after 15 s. Further hydrometer readings were taken in a doubling pattern until 48 h, resulting in a total of 16 readings. The collected data was then analysed to determine the composition of soil particles. The pH level of the soil was assessed using the method outlined by Adekiya et al.^101^. To measure the soil’s pH, 10 g of soil was placed into a clean 100 mL beaker, to which 20 mL of deionized water was added. A pH tester 20 was then inserted into the resulting suspension in the beaker, with the aim of determining the average pH from three repeated measurements. The analysis of the electrical conductivity (EC) was carried out as previously detailed Oyem and Oyem^102^. This involved adding 10 g of soil to 20 mL of deionized water and allowing it to stand for 30 min. The resulting slurry was then filtered, and the EC was measured using a Hanna digital conductivity meter. The organic carbon content was ascertained using the method reported by Mrayyan and Battikhi^103^ introducing 1 g of soil into 10 mL of 1.0 M of K_2_Cr_4_O_7_ and the mixture was shaken for homogeneity. Later, 20 mL of 98% H_2_SO_4_ was rapidly added using a burette and shaken with vigour for 1 min and left standing on a white tile for 30 min. The mixture was then added with 200 mL of deionized water and later with 10 mL of 85% H_3_PO_4_, 0.2 g NaF and 15 drops of diphenylamine indicator. The ensuing solution was back-titrated with 0.5 N iron(II) sulphate and organic carbon calculated^104^.

The Kjeldahl method was used to determine the total nitrogen content. Initially, 10 g of the sample was weighed and placed into a 500 mL Kjeldahl flask. Then, 20 mL of deionized water was added to the flask, which was shaken for a few minutes and left to stand for 30 min. Copper and sodium sulphate (1.5 g each), along with 30 mL of concentrated sulphuric acid, were added and mixed until homogeneous. The contents of the flask were heated until no froth was visible. The mixture was then boiled for 5 h, cooled, and 100 mL of distilled water was added to the flask. A boric acid indicator (50 mL) was used to rinse the sandy residue, which was then added to a conical flask positioned under the condenser of the distillation setup. The Kjeldahl flask, including the digest, was connected to the distillation unit. Sodium hydroxide (150 mL of 10 M NaOH) was added to the distillation flask and distilled until 150 mL of distillate was collected. The nitrogen content/concentration was calculated using a titration technique with a 0.01 M sulphuric acid distillate. The endpoint was indicated by a colour change from green to pink. A blank titration was also performed to obtain the blank titre^104^. The total nitrogen was calculated using Eq. (1).

1 $$\%N=Consumption-Blank \times 1.4007\times n\times \frac{100}{sample} \, size$$

where n represent normality of acid.

### Determination of total petroleum hydrocarbons (TPH)

Two grams of soil sample were heated at 50 °C and crushed well afterwards. Ten millilitres (10 ml) of dichloromethane (Sigma Aldrich, USA) was then added to the finely crushed soil and shaken firmly. To precipitate the soil, it was centrifuged at 3000×*g* for 10 min^105^. The solvent phase was removed. The TPH analysis was carried out following steps earlier prescribed by^106^. In summary, the hydrocarbon portion was stirred for 5 mins and separated using a Whatman filter paper No. 42. The extracted hydrocarbon was concentrated to 1 mL after being evaporated in a water bath. The TPH was determined using a GC spectrometer (Thermo Scientific™ Nicolet iCS). The samples were run in triplicate. The procedural blank was determined by going through the extraction and clean-up procedures using glass beads instead of a soil sample.

### Next-generation sequencing for metagenomic analyses

#### Metagenomic DNA extraction

**Figure 4:**
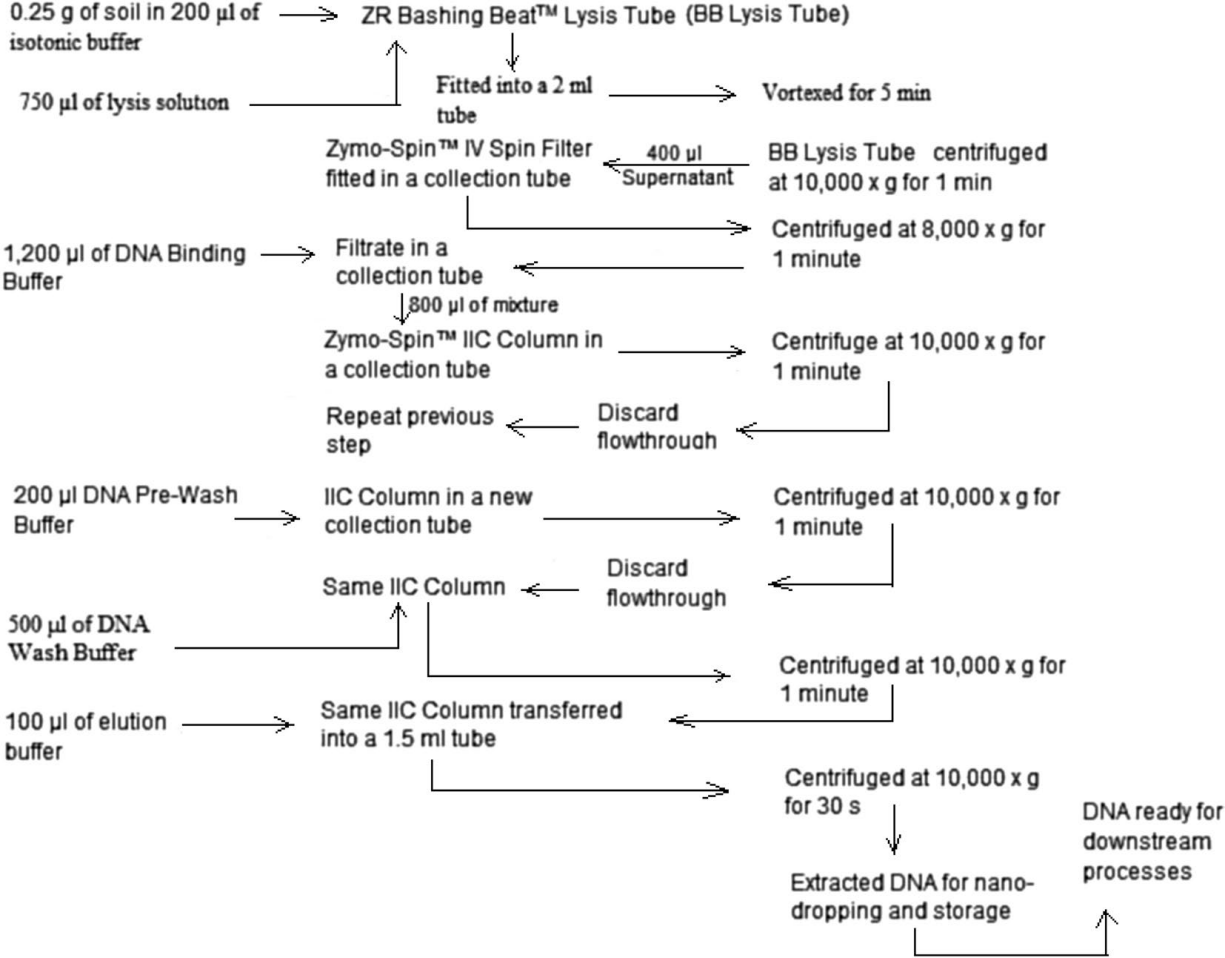
Flow diagram of community DNA extraction.

The DNA extraction was carried out on the samples using Zymo Research (ZR) Fungi/Bacteria DNA MiniPrep™ (California, USA) supplied by Inqaba Biotec, South Africa according to the manufacturer’s instructions. The summary of the extraction process is illustrated in Fig. 4. In summary, 0.25 g of soil is added to a ZR BashingBead™ Lysis Tube along with 750 μl of Lysis Solution. The tube is then processed in a bead beater at maximum speed for at least 5 min. Following centrifugation at 10,000×*g* for 1 min, up to 400 μl of the supernatant is transferred to a Zymo-Spin™ IV Spin Filter, and after centrifuging at 8000×*g* for 1 min, the filtrate is combined with 1200 μl of Fungal/Bacterial DNA Binding Buffer. Subsequently, 800 μl of this mixture is loaded onto a Zymo-Spin™ IIC Column and centrifuged at 10,000×*g* for 1 min, with a repeat of the step. The Zymo-Spin™ IIC Column is then treated with 200 μl of DNA Pre-Wash Buffer and centrifuged for 1 min at 10,000×*g*, followed by the addition of 500 μl Fungal/Bacterial DNA Wash Buffer and another round of centrifugation. The Zymo-Spin™ IIC Column is transferred to a clean 1.5 ml microcentrifuge tube, and 100 μl of DNA Elution Buffer is added directly to the column matrix. The elution is achieved by centrifuging at 10,000×*g* for 30 s, resulting in the extraction of DNA suitable for downstream analysis^107^.

#### Polymerase chain reaction (PCR)

For the PCR analysis, the hypervariable region (V3-V4) of the 16S rRNA was targeted using bacteria-specific primers, namely 341F and 806R. The primers were tagged according to^108^. All the PCRs were done in triplicate (n = 3). Polymerase chain reaction master mix aliquot was dispensed into PCR tubes and the different DNA samples were introduced into each tube alongside a negative control. The PCR reagents in each tube amounted to 50 μl containing: buffer (5 μl: 100 mM), MgCl_2_ (1.5 μl: 25 mM), universal primer1 (2 μl forward: 20 μM), primer2 (2 μl reverse: 20 μM), dNTP mix (1 μl: 200 μM), Dream Taq DNA Polymerase (0.25 μl: 1.25 units/50 μl), sterile water (35.25 μl) and DNA samples (3 μl)^109–111^. The PCR condition was set at 3 min at 94 °C, followed by 35 cycles comprising 30 s at 94 °C, 30 s at 58 °C, and 1 min at 72 °C^61^. The process concluded with a final extension step of 10 min at 72 °C. The PCRs were performed using an MJ Mini thermal cycler (Bio-Rad, Hercules, CA, USA).

The resulting amplicons were separated electrophoretically with 1% agarose gel stained with 0.1 μg/ml ethidium bromide running at 80 V for 60 min, using TAE electrophoresis buffer. The PCR amplicons were visualized by UV fluorescence to determine the amplicon sizes. The PCR products (20 μl each) were later cleaned up using 160 μl of 13% polyethene glycol (PEG) 8000, 20 μl of 5 M NaCl solution and 200 μl of 70% ethanol.

#### MiSeq sequencing and sequence analysis

The PCR products (after purification using Omega, Bio-Tek and quantification with Agilent Bioanalyzer 2100) were sequenced with the Miseq platform at the University of South Africa (UNISA), Science Campus, Florida, Roodepoort. This process involved 600 cycles (300 cycles for each paired read and 12 cycles for the barcode sequence) as per the manufacturer’s guidelines. This also involved 600 cycles (300 cycles for each paired read and 12 cycles for the barcode sequence) following the manufacturer’s instructions. The sequence data was analysed using the 16S-based metagenomics workflow provided by MiSeq Reporter v2.3 (Illumina). The 16S rRNA gene, a frequently targeted region, was used for microbial identification, thereby eliminating the need to sequence the entire genome. The Illumina workflow began with purified genomic DNA, where primers were extended with sequences that included indexing barcodes. The samples were then merged into a single library and sequenced on the Illumina MiSeq platform, resulting in paired 230 bp reads^112,113^.

#### Bioinformatic analyses

Demultiplexed paired-end reads obtained from the sequencing facility were quality-checked using FastQC software version 0.11.5 (Babraham Institute, United Kingdom). Subsequently, Trimmomatic software (version 0.38)^114^ was used to quality-trim paired reads, including clipping off any Illumina barcodes and eliminating reads with an average quality score (Phred Q score) lower than 20. Quality-filtered paired reads were then analyzed in the Quantitative Insights into Microbial Ecology (version 2) (QIIME2) software^115^. DADA2 denoiser^116^ was used to merge pair-end sequences into full-length sequences as well as remove chimaras. USEARCH version 7 was used to cluster similar sequences into operational taxonomic units (OTUs) at 97% similarity^117^. Taxonomic classification of the clustered OTUs was performed against the RDP classifier^118^. The obtained OTU table was further rarefied to even depths of 7544 sequences. The OTU and sequences of clustered OTUs were used as an input to PICRUSt2 software (installed as a QIIME2 plugin) to predict metabolic functions^119^ based on 16S rRNA. PICRUSt2 was developed in 2020 as an improvement over the 2013 version. It is more accurate and features a larger database. PICRUSt2 is a promising tool with the potential for various research applications. For instance, it could be employed to investigate the functional potential of microbial communities in different environments, as demonstrated in this study. The bacterial communities’ relative abundance was visualized at the phylum and genus level to better convey the biological information in these samples. The OTU table with assigned taxonomy was taxonomy was normalized (relative abundance) using MicrobiomeAnalyst^120^; and used to plot 100% stacked bar graph.

#### Statistical analysis

QIIME2 output—OTU table was in text and biom format. OTU table in text format was imported into Rstudio and ranacapa (ranacapa::runRanacapa()) package was used for rarefication curve, Shannon index and Observed OTUs calculations^121^ The biom format of the OTU table was uploaded to MicrobiomeDB—A data-mining platform for interrogating microbiome experiments was used to determine the top 10 abundant genera. These genera were then compared between the samples where significant (q-value > 0.05) features between two groups (HP-MP, HP-CS and CS-MP) calculated using White’s non-parametric test with Benjamini–Hochberg FDR (false discovery rate) in STAMP. The same test was used for PICRUSt2 predicted function.

## Data Availability

The metagenomic datasets generated and analysed during the current study are available in the National Centre of Biotechnology Information (NCBI) Sequence Read Archive (SRA) database under the accession number PRJNA563161 for public access.
